# Antimitochondrial M2 Antibody-Positive Myositis Presenting With Headache As the Chief Complaint: A Case Report

**DOI:** 10.7759/cureus.94964

**Published:** 2025-10-20

**Authors:** Yuriko Sato, Makoto Takahashi, Kenta Takahashi, Ryo Itabashi, Tetsuya Maeda

**Affiliations:** 1 Neurology and Gerontology, Iwate medical university, Iwata, JPN; 2 Neurology and Gerontology, Iwate Medical University, Iwate, JPN

**Keywords:** antimitochondrial m2 antibody, headache, myositis, non-invasive positive pressure ventilation, type 2 respiratory failure

## Abstract

Antimitochondrial M2 antibody (AMA-M2)-positive myositis is a muscle disease characterized by the presence of serum AMA-M2, which primarily manifests as weakness and atrophy of the trunk muscles, often accompanied by other symptoms such as weight loss, cardiac dysfunction, arrhythmia, and respiratory failure. To date, however, headache has not been reported as an associated symptom. Herein, we present the case of a 46-year-old woman with AMA-M2-positive myositis who was unaware of any notable muscle weakness and mainly complained of headaches. Based on the patient’s history and the nature of the headaches-nonpulsatile, constricting, without nausea, and unaffected by position-, a tension-type headache was suspected. However, examination in the supine position revealed a reduction in blood oxygen concentration and trunk muscle weakness, leading to an eventual diagnosis of AMA-M2-positive myositis with type 2 respiratory failure. The patient’s headache rapidly improved with the initiation of non-invasive positive pressure ventilation (NPPV), and the cause of the headache was identified as CO_2_ retention due to type 2 respiratory failure. In addition, glucocorticoid-based immunotherapy contributed to gradual improvements in weight, muscular strength, and respiratory function, thereby enabling the patient to be weaned off NPPV after 13 months of treatment. This case highlights the importance of considering neuromuscular diseases, including AMA-M2-positive myositis, in the differential diagnosis of headache and emphasizes the necessity of detailed examinations, including those conducted in the supine position.

## Introduction

Antimitochondrial M2 antibody-positive myositis, which comprises 2.5%-19.5% of all myositis cases, is characterized by serum antimitochondrial M2 antibody (AMA-M2) positivity [1,2]. Initially identified in patients with primary biliary cirrhosis (PBC) showing symptoms of muscle deficiency [3], it is now recognized as a muscular disease affecting those with or without PBC who are AMA-M2 positive [1]. Key symptoms include progressive muscular weakness and atrophy, primarily in the trunk [1,2,4]. Reports also mention cardiac dysfunction, arrhythmias [1,2,4], weight loss [2], and respiratory failure due to weakness of the respiratory muscles [2,4,5]. Notably, however, headache has not been documented as a symptom associated with this condition. Herein, we present a case of AMA-M2 myositis in which the patient’s chief complaint was a tension-type headache that improved rapidly in response to appropriate treatment, including ventilation support.

## Case presentation

A 46-year-old woman with a history of childhood appendicitis and no regular medications visited our hospital with complaints of headaches and fatigue. She had no family history of neuromuscular diseases and raised two healthy children. She worked on a poultry farm and managed her duties despite stress associated with workplace relationships.

Sixteen months before her visit, the patient experienced a weight loss of approximately 6 kg within a period of one month, whereas 13 months before the visit, she developed severe occipital headaches and morning fatigue, and the following month started to experience perceptible fatigue when climbing stairs. At 10 months before the visit, a psychiatrist had prescribed antidepressants, although she subsequently discontinued the medication due to a lack of any appreciable improvement. Given the persistent nature of the headaches and fatigue, she was eventually referred to our department by her primary care physician.

The patient visited our hospital walking unaided, was conscious and calm, and had no serious conditions requiring immediate hospitalization or acute care. The patient was notably thin with a height of 154 cm and a weight of 33 kg, although he had no visible abnormalities. Whereas blood pressure and heart rate were both within the normal ranges, her SpO2 level was slightly low at 92%. The headache was non-pulsatile, with a tightening sensation on both sides of the head; however, no nausea, jolt accentuation of headaches, sensitivity to light or sound, or signs of meningeal irritation were observed. Similarly, there was no obvious evidence of muscular weakness or atrophy in the extremities, and the patient had a normal gait and could squat. In addition, tendon reflexes were normal, and no sensory disturbance was noted. However, when placed in the supine position, thoracic respiratory motion was weak, and SpO2 levels dropped to 89%. Manual muscle testing revealed grade 3 strength and muscle atrophy in the cervical muscles, and grade 4 weakness with mild atrophy in the iliopsoas. Moreover, she was unable to perform trunk flexion using her abdominal muscles.

**Figure 1:**
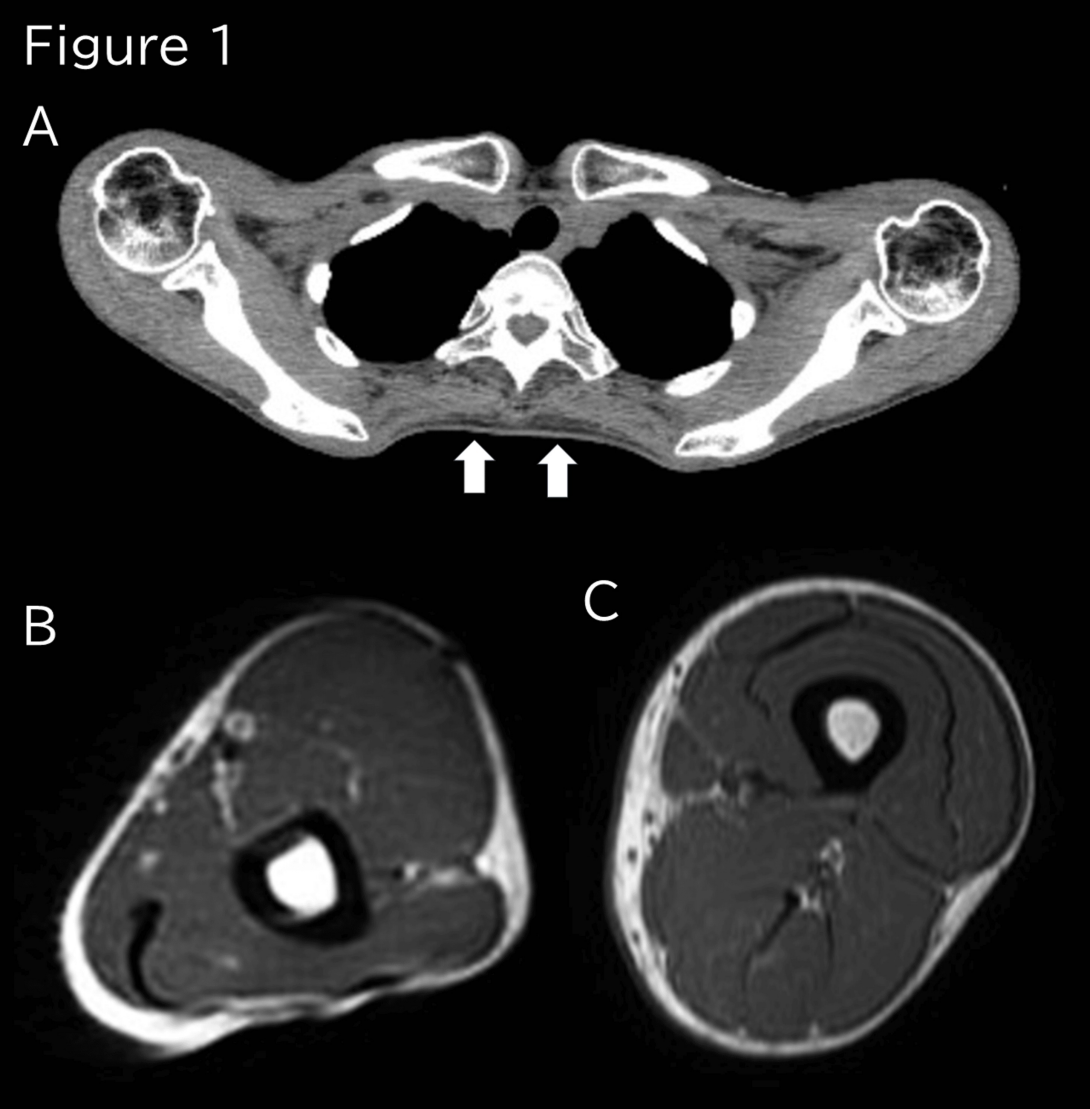
X-ray CT and MRI findings of skeletal muscles. X-ray CT provided evidence of truncal muscle atrophy (A, arrow). T2-WI of the upper (B) and lower limbs (C) showed no atrophy. T2-WI; T2 weighted magnetic resonance imaging

Blood gas analysis revealed respiratory acidosis due to type 2 respiratory failure, with a pH of 7.35, pCO2 of 73 Torr, pO2 of 62 Torr, and HCO3- of 39 Eq/L. Although blood counts were normal, blood biochemical analyses revealed slightly elevated levels of creatine kinase (CK) at 193 U/L (normal range: 41-153 U/L). There were, however, no other abnormal findings, including those for hepatobiliary enzyme levels. Similarly, tests for thyroid function, collagen disease-related autoantibodies, and myositis-specific autoantibodies were all negative, and the results of a cerebrospinal fluid examination indicated no abnormalities. Furthermore, chest X-ray computed tomography (CT) indicated no obvious abnormalities. Respiratory function tests revealed a 35% reduction in vital capacity, and although echocardiography and Holter ECG revealed no notable abnormal findings, X-ray CT and magnetic resonance imaging (MRI) of the skeletal muscles provided evidence of truncal muscle atrophy (Figure 1).

Based on these collective findings, the patient was admitted with type 2 respiratory failure associated with a neuromuscular disease.

**Figure 2:**
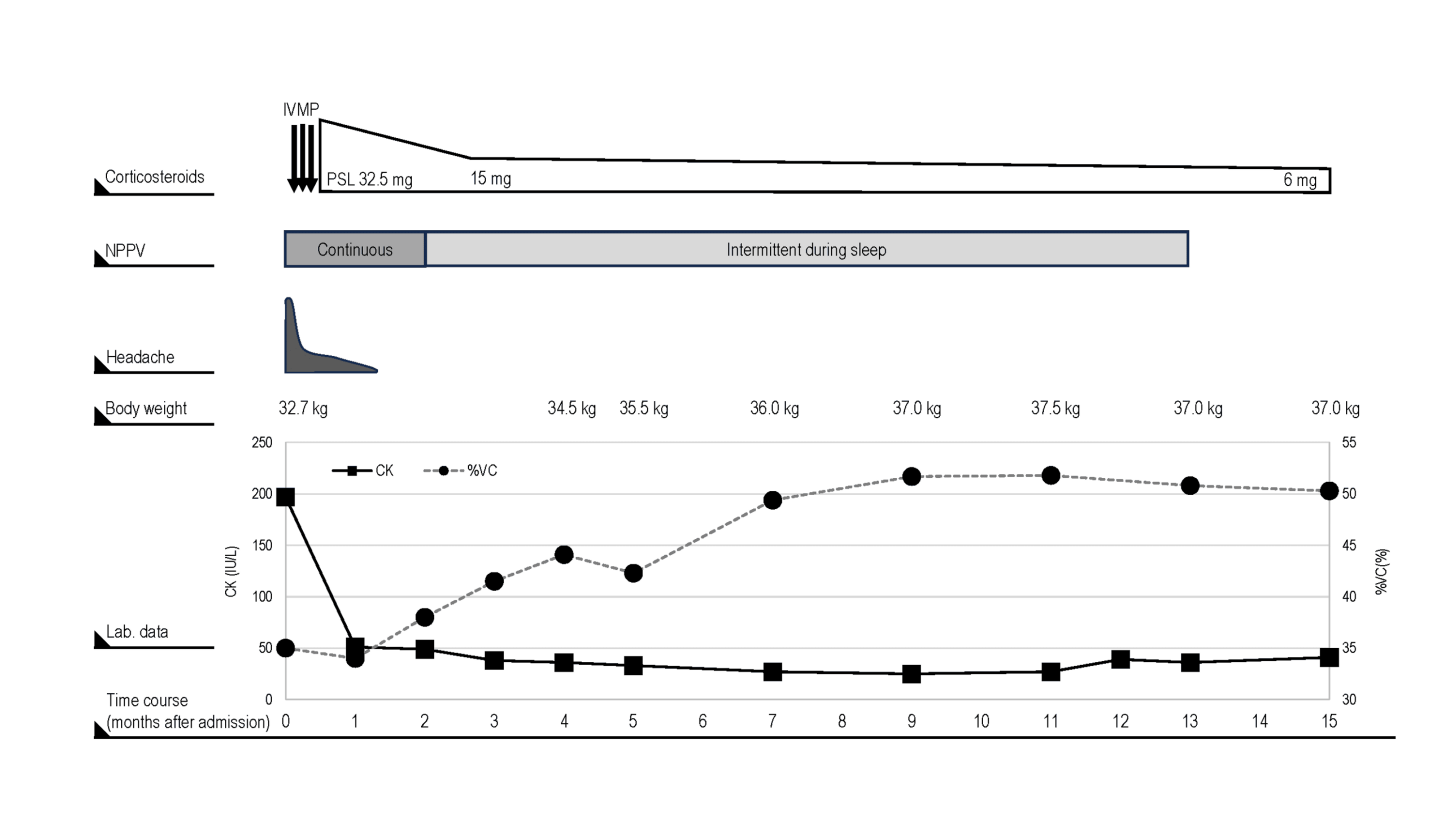
Clinical course showing treatment and changes in patient symptoms and data. Following the initiation of corticosteroids and NPPV, the headache resolved rapidly, and body weight gradually increased. Additionally, serum CK levels normalized promptly (solid line), and %vital capacity gradually improved (dotted line). IVMP: intravenous methylprednisolone, PSL: prednisolone, NPPV: noninvasive positive pressure ventilation, %VC: % vital capacity, CK: creatine kinase.

Upon admission, non-invasive positive pressure ventilation (NPPV) was initiated with the patient’s consent, resulting in a rapid alleviation of her headache. Differential diagnoses included amyotrophic lateral sclerosis (ALS), Pompe disease, sporadic late-onset nemaline myopathy (SLONM), and AMA-M2-positive myositis. Needle electromyography was performed on the lingual muscle, biceps brachii, first dorsal interosseous, vastus lateralis, and tibialis anterior muscles, revealing no obvious abnormalities. However, we were unable to perform a similar assessment of the paraspinal muscles, as the patient could not safely lie face down owing to respiratory difficulties. A muscle biopsy of the biceps revealed no specific findings, although a moderate variation in fiber size was observed. Blood analyses indicated that neutrophil alpha-glucosidase activity and protein fractions were within the respective normal ranges, although serum AMA-M2 levels were elevated at 395.1 (reference values: 0-7.0). Based on these analyses, the patient was diagnosed with AMA-M2-positive myositis and was initially treated with 1000 mg intravenous methylprednisolone for three days, followed by 32.5 mg/day oral prednisolone (PSL), which contributed to a rapid normalization of CK levels. Over the following days, the PSL dose was gradually reduced, and there were gradual improvements in the patient’s weight and respiratory function, along with a reduction in pCO2 levels. Having received treatment for 30 days, the patient required NPPV support only at night and was completely weaned off the support after 13 months of treatment with 6 mg/day PSL (Figure 2).

## Discussion

In this case study, we diagnosed a patient with AMA-M2-positive myositis and associated type 2 respiratory failure. Our findings in this case highlight the following three key points: the confirmed diagnosis of AMA-M2-positive myositis; the possibility that headache, rather than muscular weakness or respiratory distress, may be the primary complaint; and the efficacy of immunotherapy in alleviating the multiple symptoms of AMA-M2-positive myositis.

Although there are currently no established diagnostic criteria for AMA-M2-positive myositis, the diagnosis relies on characteristic clinical symptoms and laboratory findings indicating positivity for serum AMA-M2, as well as the exclusion of other differential diseases. Patients typically present with muscular weakness and atrophy in the trunk compared with the extremities [1,2,4], along with weight loss, respiratory failure, cardiac dysfunction, and arrhythmia [2-5]. With the exception of the absence of cardiac-related symptoms, our findings in this case are consistent with these characteristic symptomatic. Serologically, elevated levels of CK exceeding 1000 IU/L are often observed in AMA-M2-positive myositis [1,2,4], although cases with normal levels have also been reported [2]. The CK levels recorded in the patient described herein were only slightly elevated, reaching a maximum value of 193 U/L, and were effectively reduced to 30 U/L in response to treatment with glucocorticoids. No hepatobiliary enzyme abnormalities were detected, and PBC is not always required for this diagnosis [1,2,4]. Both X-ray CT and MRI revealed trunk muscular atrophy, although in the absence of any abnormal signals, and a muscle biopsy revealed non-specific findings with moderate fiber size variation, which is consistent with AMA-M2-positive myositis [1]. As differential diagnoses, we initially considered other neuromuscular diseases, such as ALS [6,7], Pompe disease [8], and SLONM [9], which can cause type 2 respiratory failure despite the ability of those affected to walk, although we obtained no supportive evidence indicative of any of these. However, our diagnosis of AMA-M2-positive myositis was supported by the fact that the patient’s symptoms improved in response to immunotherapy.

A notable feature of this case is that the patient’s main complaint was that of headaches, as opposed to muscle weakness or respiratory distress, which is a symptom that hitherto has not been reported in association with AMA-M2-positive myositis. The headaches experienced by this patient resembled a tension-type headache, characterized by a strangling sensation without pulsation, nausea, vomiting, jolt accentuation, and sensitivity to sound or light. It was most severe upon waking and improved in response to the initiation of NPPV, which is consistent with the criteria for sleep apnea headaches specified in the International Classification of Headache Disorders [10]. Similarly, ALS, which can lead to type 2 respiratory failure, has been associated with headaches [7]. This indicates that the headaches in this case may have been a consequence of CO2 retention due to type 2 respiratory failure. Notably, patients with this condition experience less pronounced dyspnea despite reduced lung capacity and elevated PaCO2 levels. We believe that in the present case, the patients’ symptoms began 16 months before her initial hospital visit, coinciding with the time when she started to lose weight, and that the absence of dyspnea was due to a gradual decline in respiratory function.

Similarly, the patient was largely unaware of any muscular weakness, which was not evident during the examination in either the sitting or standing position. Although initially, we suspected a tension-type headache, when the patient was placed in the supine position, we detected reductions in SpO2 levels and weakness in the neck and trunk muscles, which contributed to arriving at our eventual diagnosis. We speculated that the reduction in SpO2 levels in the supine position was attributable to the effects of gravity restricting thoracic movement. Consequently, to avoid overlooking the possibility of AMA-M2-positive myositis, thorough examinations, including assessments in the supine position, are essential, even in patients with tension-type headache-like symptoms.

A key finding in this case was the improvement of symptoms in response to immunotherapy. Although there is currently no standard treatment for AMA-M2-positive myositis, several reports have highlighted the advantages of immunotherapy. Glucocorticoid-based immunotherapy has been shown to improve serum CK levels, muscle weakness [4], activities of daily living [1,2,4], weight loss [2], respiratory function [2,5], and cardiac symptoms [2,3]. In the present case, we consistently found that intravenous and oral glucocorticoid treatment improved serum CK levels, reduced weight loss, and alleviated muscle weakness, thereby enabling a successful weaning off NPPV. Thus, AMA-M2-positive myositis is a condition for which the diverse symptoms can be ameliorated by initiating immunotherapy, and should be actively considered in the differential diagnosis.

## Conclusions

In this report, we describe the case of a patient with AMA-M2-positive myositis, for whom the primary complaint was headache, with little awareness of muscular weakness or dyspnea. Notably, the patient was initially misdiagnosed based on the findings of sitting- and standing-based assessments, as the headache resembled a tension-type headache and muscular weakness was not apparent. In evaluating patients presenting with headache as the primary complaint, it is important to consider type 2 respiratory failure due to neuromuscular disorders, including AMA-M2-positive myositis, as part of the differential diagnosis. Detailed examination in the supine position is essential for assessing trunk muscle weakness and respiratory muscle dysfunction.

## References

[1] Maeda MH, Tsuji S, Shimizu J (2012). Inflammatory myopathies associated with anti-mitochondrial antibodies. Brain.

[2] Nagai A, Nagai T, Yaguchi H (2022). Clinical features of anti-mitochondrial M2 antibody-positive myositis: case series of 17 patients. J Neurol Sci.

[3] Uhl GS, Baldwin JL, Arnett FC (1974). Primary biliary cirrhosis in systemic sclerosis (scleroderma) and polymyositis. Johns Hopkins Med J.

[4] Hou Y, Liu M, Luo YB (2019). Idiopathic inflammatory myopathies with anti-mitochondrial antibodies: clinical features and treatment outcomes in a Chinese cohort. Neuromuscul Disord.

[5] Fujii S, Horiuchi K, Oshima Y (2021). Inflammatory myopathy associated with anti-mitochondrial antibody presenting only with respiratory failure. Intern Med.

[6] Feldman EL, Goutman SA, Petri S, Mazzini L, Savelieff MG, Shaw PJ, Sobue G (2022). Amyotrophic lateral sclerosis. Lancet.

[7] Shoesmith CL, Findlater K, Rowe A, Strong MJ (2007). Prognosis of amyotrophic lateral sclerosis with respiratory onset. J Neurol Neurosurg Psychiatry.

[8] Menzella F, Codeluppi L, Lusuardi M, Galeone C, Valzania F, Facciolongo N (2018). Acute respiratory failure as presentation of late-onset Pompe disease complicating the diagnostic process as a labyrinth: a case report. Multidiscip Respir Med.

[9] García Estévez DA, Juanatey-García A, San Millán Tejado B, Barros Angueira F (2024). Late-onset sporadic nemaline myopathy presenting as hypercapnic respiratory failure. Neurologia (Engl Ed).

[10] Headache Classification Committee of the International Headache Society (IHS) (2013). The International Classification of Headache Disorders, 3rd edition (beta version). Cephalalgia.

